# Uncovering Enzyme-Specific Post-Translational Modifications: An Overview of Current Methods

**DOI:** 10.3390/proteomes13030037

**Published:** 2025-08-11

**Authors:** Nashira H. Ridgeway, Kyle K. Biggar

**Affiliations:** Institute of Biochemistry, Carleton University, Ottawa, ON K1S5B6, Canada; nashiragrigg@cmail.carleton.ca

**Keywords:** post-translational modification, proteomics, protein function, cellular signaling

## Abstract

Post-translational modifications (PTMs) govern a multitude of protein functions within the cell, surpassing the basic function(s) encoded directly within the amino acid sequence. Despite the historical discovery of PTMs dating back over a century, recent technological advancements have facilitated the rapid expansion of the known PTM landscape. However, the elucidation of enzyme–substrate relationships responsible for PTMs, particularly for those less studied, remains a challenging endeavor. This review provides an extensive overview of methods employed in the discovery of enzyme-specific substrates for PTM catalysis. Beginning with traditional experimental approaches rooted in chemistry, biochemistry and cell biology, this review progresses to recently developed computational strategies tailored for identifying enzyme–substrate interactions. The analysis reflects on the remarkable progress achieved in PTM research to date, underscoring the increasing role of computational and high-throughput techniques in expediting enzyme–substrate discovery. Furthermore, it highlights the potential of artificial intelligence to revolutionize PTM research and emphasizes the importance of unbiased high-throughput analysis in advancing our understanding of PTM networks. Ultimately, the review advocates for the integration of sophisticated computational strategies with experimental techniques to unravel the complex enzyme–substrate networks governing PTM-mediated cellular processes.

## 1. Introduction

In the intricacies of cellular regulation, post-translational modifications (PTMs) emerge as the orchestrators of a myriad of protein functions beyond the basic sequence of amino acids (AAs). Many of these small modifications occur within the side chain (e.g., -R group) of an AA residue within a protein, potentially drastically altering cellular machinery and function.

**Figure 1:**
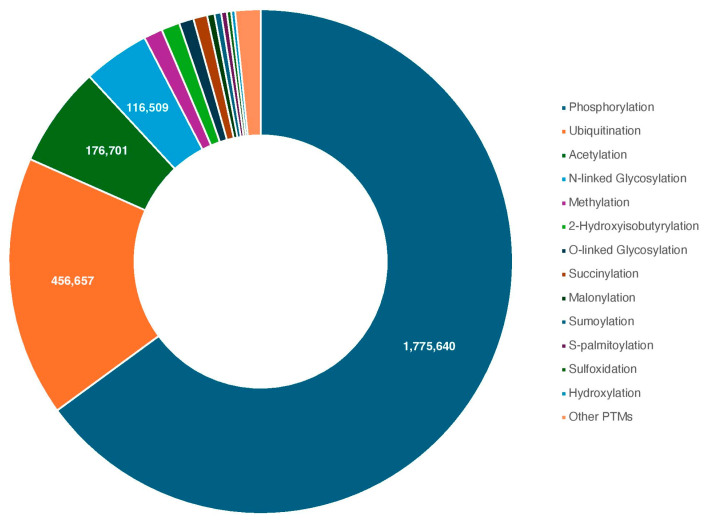
Human PTMs discovered to date, represented graphically. Each PTM is color coded, with numerical descriptions of the four largest: phosphorylation, ubiquitination, acetylation, and N-linked glycosylation. Other PTMs are compiled into one portion. Compiled from the dbPTM database [6].

Historically, the very first PTM was uncovered by Levene and Alsberg in 1906, with the discovery of phosphorylated serine within the protein, vitellin [1]. This discovery predated the identification of all 20 essential AAs, and was assumed to be a novel addition until an investigation revealed it was a modified serine in 1932 [1,2]. As technology improved, so did the techniques to uncover novel PTMs, rapidly populating the list of known PTM substrates. To date, over six hundred unique classes of PTMs have been detected within the proteome through high-throughput experiments [3,4]. Despite the relatively large variety of modifications documented, just three PTMs account for over 90% of reported PTM activity, namely phosphorylation, acetylation, and ubiquitination (Figure 1) [3]. The initial discovery of a previously unknown modification is followed by the detailing of its cellular relevance and mechanisms underlying the function of the PTM. Currently, the majority of PTM classes are mediated by families of enzymes that specifically recognize modification sites with a high degree of specificity [5]; for example, there are classes of enzymes (e.g., Su(var)3-9, Enhancer-of-zeste and Trithorax (SET)-domain containing lysine methyltransferases) that mediate a large number of lysine methylation events. Although implicated in both disease and cancer, these PTM-inducing or protein-modifying enzymes are a relatively underexplored field, with many enzymes possessing just a handful of characterized protein substrates.

In this review, we will present a comprehensive overview of methods employed in the discovery of substrates for the enzymes responsible for PTM catalysis. Commencing with traditional *in vivo* and *in vitro* approaches, we will later delve into computational strategies tailored for enzyme-specific substrate identification.

## 2. PTMs and the Enzymes Responsible

**Figure 2:**
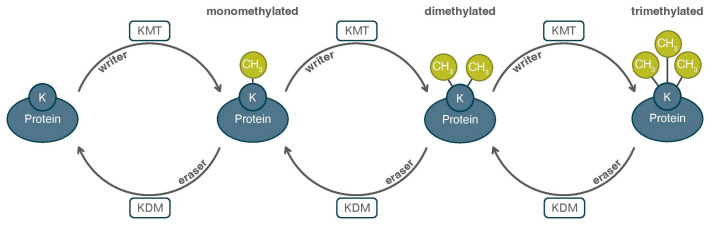
The dynamic nature of PTMs, exemplified by lysine methylation. Lysine residues may be mono-, di-, or trimethylated by methyltransferases (or “writers”), with the removal of each group catalyzed by demethylases (“erasers”).

The dynamic lifecycle of a reversible protein PTM typically involves its addition or removal to/from AA residues within proteins. These are typically small molecules, such as methyl, acetyl, or phosphoryl groups among many others. As mentioned, over 600 different modifications have been observed through high-throughput analytic methods, and many of these are known to be reversible [3]. A PTM can broadly lead to several outcomes directing the function and cellular relevance of the modified protein. Additionally, the dynamic nature of PTMs make them a contributing factor in proteoforms, as their presence or absence throughout a protein can contribute to a protein’s many alternative states. For example, a PTM may alter the structure of a protein, or affect how it interacts with other proteins and cellular mechanisms [3]. A single AA may display a specific PTM within a protein, or many, and often multiple PTMs can collectively mediate protein function or exert influences on neighboring modifications (e.g., PTM crosstalk) [7]. As an example, lysines are a heavily modified residue, subject to acetylation, methylation, succinylation, and malonylation, amongst others [8]. The enzymes that create or remove PTMs can be simply defined as “writers” and “erasers”, respectively. For example, in the case of lysine methylation, the modifying enzymes are lysine methyltransferases (a.k.a. writers) and lysine demethylases (a.k.a. erasers) (Figure 2). More broadly, the writing or erasing of a PTM by these enzymes directly links them to proteoform diversity.

As previously discussed, PTMs have the potential to influence various aspects of protein function, including enzyme activity and assembly, protein stability and interactions, cellular communications, molecular trafficking, as well as protein solubility, folding, and localization [3]. Many investigations have implicated certain PTMs with key cellular functions and the adaptability of the cell in response to environmental changes [9]. To date, the aims of PTM identification have focused towards “important” or highly abundant proteins, such as secretory, membrane, and histone proteins, however increasing investigations demonstrate the benefits of uncovering the full scope, and complexity, of PTM activity within the proteome [3].

Although the full extent to which PTMs occur within the proteome remains under active investigation, recent work has focused on the mediators of PTMs. The majority of PTMs are enzyme-mediated; however, some exist through nucleophilic or redox-sensitive -R groups meeting particularly reactive metabolites [5]. Some examples of non-enzymatic PTMs include protein oxidation and protein acylation [5]. As reflected in the nomenclature, enzyme-mediated PTMs involve additional machinery, namely, enzymes to facilitate modification dynamics—a key component being the ability of an enzyme to recognize a specific substrate. Commonly referred to as a “recognition motif”, this is a sequence of AAs that an enzyme requires to recognize a substrate and enact a PTM. Cofactors are also required by enzymes, such as S-adenosyl-L-methionine (SAM), which supplies the required sulfonium methyl group for lysine methyltransferases (KMTs) [10]. Often, PTM-writing enzymes are themselves regulated by PTMs that function to regulate the enzyme itself. This careful choreography may be observed within the intricate network of known methyltransferase substrates [11]. The interplay between enzymes, substrates, and other enzymes is a frequent area of study (e.g., functional proteomics) as PTM-inducing enzymes are often over- or under-active in disease states.

Historically, the investigation into the origins of PTMs lagged behind their initial discovery, as the prevailing belief was that each PTM represented a distinct AA [2]. Eventually, as the 20 essential AAs were confirmed, investigations probing the enzymes responsible for these modifications were subsequently underway. The first class of enzymes to be probed for its effect on protein substrates were kinases, a class of enzymes that mediate protein phosphorylation. In 1954, Burnett and Kennedy demonstrated the phosphorylation of a protein substrate with a radioactively labeled cofactor, adenosyl triphosphate (ATP) [12]. This protein substrate was later discovered to be the enzyme glycogen phosphorylase (GP), and the phosphorylation was completed by a kinase, aptly named phosphorylase kinase (PhK) [13,14,15]. The unravelling of the biological importance behind this paradigm later came in 1969, where it was demonstrated that this regulatory protein phosphorylation event affected the enzymatic activity of GP, which participates in cellular metabolism [16]. The next key enzyme–substrate mechanism found was the acetylation and methylation of histone proteins by Allfrey, Faulkner, and Mirsky in 1964 [17]. This key finding not only clarified that the modifications occurred post-translation, but it also suggested that histones had a role in the transcription of RNA, and hence mRNA expression [17]. These significant findings were the first of many linking PTMs to enzymes, which improved with the rapidly evolving technology.

The overwhelming representation of phosphorylation within the PTMs discovered to date (Figure 1) is likely due to several factors, the first being its sheer categorized abundance within the cell, likely linked to its wide involvement in cellular processes, from proliferation to apoptosis [18]. Additionally, the family of protein kinases, the enzymes responsible for phosphorylation, is one of the largest within eukaryotes [18]. Furthermore, as protein phosphorylation was the first discovered PTM, techniques to uncover sites of phosphorylation and the enzymes responsible are highly represented within the literature [3,16,19,20]. As prominent negatively charged modifications, much of the chemistry applied to isolating and detecting the phosphate group does not translate to other PTMs, hence new techniques are constantly under development, as this review will detail, increasing the representation of other PTMs within the landscape.

Comprehending the method by which each enzyme functions allows for critical insight into cellular mechanisms. Many protein-modifying enzymes act upon important cellular machinery such as histones, but also play diverse roles in other vital pathways. One such example is the KMT SET8 (a.k.a. SETD8, KMT5A and PR-SET7), responsible for the mono-methylation of lysine 20 within histone H4, as well as lysine 382 of the p53 tumor suppressor, reducing p53-mediated transcription of target genes [21]. Another well-documented example is the lysine deacetylase SIRT2, known to deacetylate lysine 16 of histone H4, but also α-tubulin at lysine 40 [22].

Detailing the full extent to which an enzyme enacts PTMs can inform the development of therapeutics towards disease. Accordingly, many PTM-modifying enzymes have become therapeutic targets due to their overactivity in times of cellular stress. The first approved therapeutic targeting a PTM-modifying enzyme was Imatinib (Gevec^®^) in 2001, a c-Abl tyrosine kinase inhibitor developed for the treatment of chronic myeloid leukemia [23]. The involvement of PTM-inducing enzymes in vital cellular processes such as apoptosis, protein–protein interactions, cell cycle control and cell signaling often implicates them in disease and cancer. Beyond their heavy involvement in cancer, PTM-inducing enzymes have been implicated in diabetes (type 1 and type 2), nervous system diseases, and cardiovascular diseases [23]. A broad implication of PTMs has been associated with immune, digestive, muscular, and respiratory diseases [3]. The range of cancer and disease involvement narrows when observing a specific PTM or enzyme family. One may infer that the data forming these observations may be skewed towards the three PTMs that compose 90% of the PTM data, highlighting the need to uncover novel PTM substrates (Figure 1). This is further emphasized by recent investigations, such as those within the relatively underexplored field of SUMOylation, and the emerging space of ADP-ribosylation knowledge and actively addresses their underrepresentation within the PTM space through exploratory *in silico* and *in vitro* approaches [24,25,26]. Moreover, urPTMdb, a database of underrepresented PTMs, has been developed to specifically address the concern of under exploration within the proteomic space [27].

Given the importance of finely tuned regulation of PTM events in the cell, the dysfunction or alteration of cellular function(s) mediated by PTM-inducing enzymes is a frequent driver of cellular stress or malfunction, a common occurrence in cancers. The overabundance, under-abundance, or general dysfunction, of such enzymes often occurs in a variety of cancers [28]. This is likely due to PTM-inducing enzymes often regulating cancer-mediating responses as well as DNA repair pathways. As in disease involvement, some enzyme families are wholly implicated in a handful of cancers, though often PTM-inducing enzymes themselves are unique through their modification of substrates and hence are implicated in unique diseases and cancer. The unique PTM network surrounding the enzyme responsible is key to understanding the cellular mechanics that give rise to this observation.

## 3. *In Vitro* Approaches to Enzyme–Substrate Discovery

Often conventional approaches to studying substrate networks of PTM-inducing enzymes involve identification of the enzyme responsible for previously identified sites of modifications (e.g., bottom-up discovery). Investigations are frequently influenced by prior research into other enzyme family members which share a common catalytic domain. This is, in part, due to some methods relying on techniques that are PTM specific. It is also important to note that as these methods are often performed outside of the cell, known as *ex vivo* or *in vitro*, they are typically utilized as an exploratory precursor to the verification of a novel site of PTM activity through directed in-cell experimentation.

The earliest and most basic method to determine enzyme-substrates is simple protein extraction. In brief, isolated proteins are digested and purified, then treated with the PTM-inducing enzyme and a labelled cofactor. The presence of the label is determined through protein purification techniques, such as gel electrophoresis, among others [29]. This technique is arguably one of the most established and straightforward techniques of identifying substrates; however, it requires prior knowledge of both the enzyme and substrate, and the requirement to purify both poses a challenge, particularly so when faced with the full extent of the proteome. This method is best suited for confirmation, rather than exploration. However, this was the technique applied to uncover the first enzyme–substrate networks prior to the advent of protein/peptide array method for *in vitro* investigations [29].

In 2000, Snyder and colleagues presented the novel concept of spotting thousands of purified proteins on a microscope slide to determine substrates of protein kinases in yeast [30]. This process involved the purification of 5800 proteins with glutathione-S-transferase (GST) and polyhistidine (6xHis) tags through affinity chromatography [30]. The proteins were spotted onto a nickel-coated microscope slide to create a yeast “protein chip” [31]. A total of 87 yeast phosphorylating kinases were applied to identify over 4000 phosphorylation events, defined through the use of radioactively labelled ATP [30]. Since then, protein microarrays have become commercially available and applied to study a variety of PTMs, including ubiquitin ligases, tyrosine phosphatases, lysine acetyltransferases, KMTs, and more [32,33,34]. Though this high-throughput method allows for the testing of thousands of potentially PTM-modified proteins at once, they are expensive to generate and purchase. Moreover, protein microarrays do not indicate where a PTM is occurring within a protein (i.e., site of modification) or reflect upon the in-cell dynamics. The isolation of proteins on the slide prevents the formation of enzyme complexes, or the cellular microenvironments that may be required for PTM activity.

As researchers sought alternative platforms to overcome the limitations of protein microarrays—namely their cost, lack of PTM site specificity, and inability to mimic cellular context—modern peptide array-based approaches emerged as a versatile and scalable solution. Peptide synthesis was first presented with the creation of Merrifield solid-phase peptide synthesis (SPPS) in 1963 [35]. In this process, peptides are assembled on a solid resin support, AA by AA, with deprotection, activation, and coupling steps cyclically repeating until eventual cleavage [35]. The peptides generated with SPPS are generally of high quality; however, they quickly become expensive in respect to time and reagents when hundreds-to-thousands are synthesized in parallel. The mid 1980s saw the creation of in situ synthesis, in which peptide synthesis could be run in parallel, using less reagents and eliminating the requirement of peptide purification [36,37]. In 2002, SPOT synthesis was introduced by Ronald Frank [38]. F-moc protected AAs allowed for the parallel synthesis of peptides on a solid membrane support by hand [38]. Solutions containing the AAs and coupling reagents were dispensed on predefined spots, with full-membrane washing following coupling reactions to deprotect the functional groups prior to the next coupling cycle [38]. Shortly thereafter, an automated liquid handling system was developed to automate the process and minimize the reagents, such that the technique became widely available [39].

Variations on SPOT synthesis have optimized peptide synthesis to increase throughput while decreasing cost [40]. Peptide beads are another option, in which the solid support is beads rather than a membrane [41]. Other innovative techniques include particle-based synthesis, in which a 24-ink laser printer transfers toner particles containing F-moc protected AA particles onto a solid support [42]. The particles are melted to allow coupling; then, the membrane is washed, and more particles are deposited [42]. This technique is capable of synthesizing 40,000 peptide spots per square centimeter on a microchip [42]. The photolithographic method introduced by Fodor in 1991 predated SPOT synthesis, utilizing AA reagents protected with photolabile groups deprotected with irradiation [43]. Much like the other methods, deprotection is followed by the addition of the next AA prior to another round of deprotection until the AA sequence is completed [43]. Improvements in technique allowed for faster and denser generation, now capable of up to ~10 million peptides on a single slide, though highly specialized equipment is required [39,44].

**Figure 3:**
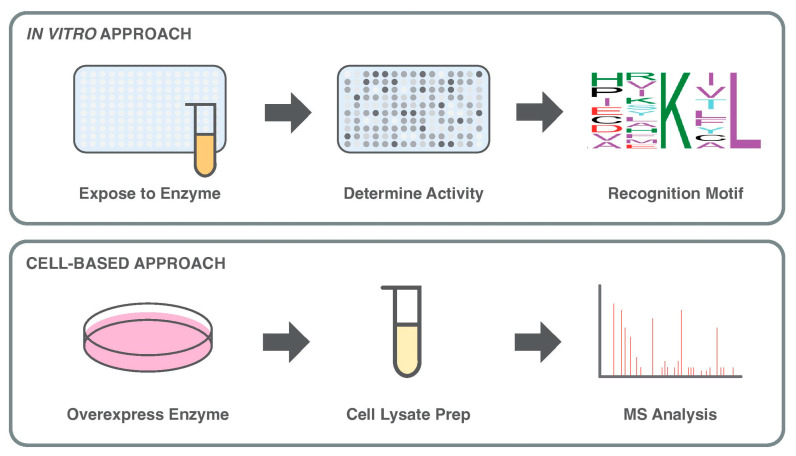
A basic, visual summary of *in vitro* approaches to enzyme-specific substrate discovery, as compared to cell-based approaches. The *in vitro* approach is represented by peptide arrays and recognition motifs, whereas the cell-based approach demonstrates the basic concepts of MS analysis.

The nature of peptide arrays is conducive to systematic investigations, such as determining the recognition motif of a PTM-inducing enzyme. In binding or recognition motif analysis, a known sequence of a substrate (e.g., peptide representation of a modification site) is systematically altered by single AA permutations throughout its sequence, typically 10–20 AAs in length (a.k.a. permutation array). Exposure of the peptide sequences to the enzyme of interest, along with a labelled probe for incorporation to the substrate(s), determines the accepted permutations in the substrate that permit enzyme activity, enabling the construction of a motif. This motif may be searched for within the proteome through various bioinformatic approaches (Figure 3) [45]. First applied to an investigation of proteases with peptide inhibitors, in 2000 Hilpert et al. applied permutations to a turkey ovomucoid inhibitor, known to inhibit the activity of porcine pancreatic elastase [46]. The mutated peptides provided insights into the binding and inhibition of porcine pancreatic elastase, which were applied to generate a novel inhibitor peptide that demonstrated both an increase in specificity and inhibitive properties [46]. Since its creation, recognition motif analysis has been often applied to investigate PTMs such as KMTs, key to the identification of non-histone substrates [47,48,49].

Oriented peptide array libraries (OPALs) are also applied to determine the dynamics of the recognition site of a PTM-inducing enzyme. OPALs differ from permutation arrays by maintaining only one AA and degenerating the rest of the sequence randomly in equal molar ratios. This defines the specificity of the recognition motif of the enzyme without requiring prior knowledge of any substrates. Similar to motifs obtained through permutation arrays, OPAL-based motifs may be applied to a bioinformatic search within the proteome for matches indicating potential novel PTM sites [50]. The OPAL technique has been applied to investigate potential substrates of tyrosine kinases, as well as arginine and KMTs, typically covering ±4 AAs from the central AA known to be modified [51,52,53]. The randomized nature of OPAL analysis is similar to the randomized library search method applied to protein kinases [54]. Here, randomized AA mixtures are created, with random substrates at just one to two positions rather than the entire sequence [54,55].

The high-throughput capabilities afforded by peptide arrays allow for large peptide libraries to be synthesized efficiently and in parallel. This is often a preliminary technique employed to detail the substrate network of PTM-inducing enzymes with little to no known substrates. To construct the library, combinatorial representations of a directed subset of the proteome may be utilized, enriched for sites exhibiting the protein of interest [56]. Alternatively, randomly generated libraries have also been successfully applied, in which peptides maintain the central AA while the rest of the sequence is randomized, though not at an equal molar ratio as in the OPAL approach [57]. Such comprehensive peptide libraries have been applied to characterize deacetylases and kinases substrates with libraries spanning from 700 to 6800 peptides in size [56,57,58,59,60]. In time, improvements upon the quality and quantity of PTM sites within databases have enabled more comprehensive peptide libraries.

Much like protein arrays, peptide arrays are also performed *in vitro*, attempting to mimic the conditions of the cellular environment in a highly simplistic manner. Unlike protein arrays, the size of peptides enables the location of a PTM to be better understood. Purification is not required, and synthesis has become cheap and accessible with the availability of automated techniques. Peptide arrays are another informative and exploratory method for substrate networks of PTM-inducing enzymes, although the protein structure of a potential substrate fails to be effectively represented by a peptide sequence. As the fully formed state of a protein heavily implicates the capacity of an enzyme to induce a PTM, peptide array experiments are often utilized as an exploratory method to investigate sites of PTM activity.

## 4. Cell-Based Approaches to Explore Enzyme–Substrate Networks

The application of tandem mass spectrometry (MS/MS) within the field of proteomics was transformed following the development of electrospray ionization (ESI). Introduced by Malcolm Dole in 1968, ESI gently ionizes macromolecules, resulting in a net positive or negative charge prior to MS/MS. John Fenn and Joichi Tanaka improved upon ESI in 2002 to sensitively detect a wide range of PTMs, a critical feat that awarded the pair the Nobel Prize [2,61]. Although ESI-MS/MS led to the identification of many new PTMs, the initial introduction did not result in a sharp increase in the rate of PTM discovery [2]. This is due to the requirement of purified and enriched samples for MS analysis, highly specific to the intent and PTM of the investigation (Figure 3). To address these limitations, different proteomic strategies were developed to enhance PTM coverage and contextual insight. Among these, bottom-up proteomics became the dominant workflow, relying on enzymatic digestion of proteins into peptides before MS analysis. While widely used, this approach can obscure the combinatorial nature of PTMs by disrupting the native structure of proteins.

In contrast, top-down proteomics offers a powerful complement by enabling the direct analysis of intact proteins and their complete array of modifications. This approach maintains the native proteoform and preserves crucial contextual information—such as PTM crosstalk and spatial proximity—that are often lost during peptide digestion. As a result, top-down strategies provide improved resolution of PTM landscapes and greater potential for mapping enzyme–substrate relationships at the proteome level. While technically demanding and limited by protein size and solubility, recent advances in instrument sensitivity and separation methods are increasingly supporting the application of top-down proteomics in complex biological systems.

Comparative analysis is commonly applied with MS methods, in which the studied PTM-inducing enzyme is affected through inhibition, knockdown, or protein overexpression. The treated samples provide an avenue for comparison with those untreated, pointing to differences within the proteome. It is possible to perform the treatment with the enzyme after affinity purification, prior to ESI-MS/MS, or the treatment may be provided *in vivo* prior to enrichment and analysis [62]. Liquid chromatography (LC) was then coupled with MS once ESI was developed, as it enabled micro-liter sample volumes to be analyzed. Since the 1990s, two-dimensional (2D) column-based LC-MS techniques have improved [63]. Often the 2D-LC methods separate by charge, then hydrophobicity. This technique is commonly used for peptide fractionation prior to MS to improve sensitivity and resolution towards PTMs [63].

The employment of PTM-specific antibodies provides another key avenue for sample enrichment and PTM purification prior to ESI-MS/MS identification, though they can pose a challenge when not considering other protein features. Several PTM-specific antibodies have been created towards phosphorylation, acetylation, myristoylation, prenylation, sumoylation, and ubiquitination [63]. The application of antibodies to trimethylated lysine residues has also been demonstrated within affinity enrichment, although mono- and dimethylation-specific antibodies typically suffer from issues of specificity, rendering them unreliable for highly specific affinity enrichment purposes [63]. Ligand affinity enrichment may be applicable to PTMs with affinity for metal or inorganic ions. This is the case for sulfated peptides, which may be separated with gallium, or phosphorylated peptides with metal ions and oxides [63]. PTMs participating in protein-ligand systems can also be exploited for affinity enrichment using the ligand. Additionally, PTMs can be labelled with tags through labelled cofactors, which may be applied in affinity enrichment [63].

To directly resolve enzyme–substrate relationships, beyond broad proteome-level comparisons, several targeted and innovative strategies have been developed. These include the use of catalytically inactive “substrate-trapping” mutants, which retain substrate binding but lack catalytic activity, allowing transient interactions to be preserved and captured for MS identification [62]. Similarly, photo-crosslinking approaches can covalently stabilize weak or short-lived interactions between modifying enzymes and their substrates, enhancing detection sensitivity in complex samples [62]. Analog-sensitive kinase alleles (ASKA), in which kinase active sites are engineered to accept uniquely labeled ATP analogs, have enabled selective labeling and identification of direct phosphorylation targets *in vivo*. In a fundamental study by Ubersax et al., this strategy was used in combination with radiolabeling to identify over 200 putative Cdk1 substrates in yeast [64].

Building on these targeted tools, proximity labeling methods such as BioID, TurboID, and APEX have recently gained traction [65,66,67]. These enzymes generate reactive intermediates that label proximal proteins with biotin in living cells. When fused to PTM enzymes, they allow for the spatial and temporal capture of substrate candidates that are near the enzyme during active catalysis. These approaches are particularly useful for mapping dynamic interactions or enzyme–substrate complexes in specific cellular compartments. Some genetically engineered methods combine trapping and labeling. For example, enzyme mutants that trap substrates can be further modified with photoactivatable residues, enabling crosslinking of bound targets upon UV exposure and facilitating downstream enrichment. These methods have been applied to a variety of enzyme classes, including kinases and E3 ligases [62].

In addition to these advanced methods, MS-based workflows often still rely on enzymatic digestion of proteins into peptides prior to detection. This bottom-up format can obscure the full context of PTM patterns and create ambiguity in site assignments, particularly when peptides are multiply modified [68]. Although essential, this step limits full sequence coverage, especially when proteolytic fragments are poorly ionized or difficult to assign confidently.

Phage display libraries offer another means of probing enzyme–substrate preferences. Here, phage-expressed peptide or protein libraries are exposed to PTM enzymes under labeling conditions (e.g., ATP analogs for kinases), and enriched clones are identified through autoradiography and sequencing [69]. While this method enables high-throughput screening, it is less informative for precise PTM site localization, and expression in bacterial systems can hinder proper folding of complex substrates. Collectively, the integration of enzyme-specific strategies—ranging from ASKA and substrate traps to proximity labeling—has enabled direct mapping of PTM enzyme–substrate networks in increasingly complex and native contexts. These methods complement global MS workflows and overcome limitations of correlation-based inference, providing mechanistic insight into PTM-driven signaling.

## 5. Modern Computational Approaches to Guide Enzyme–Substrate Discovery

Computational approaches have greatly expanded our ability to explore the post-translational modification (PTM) landscape, but they remain constrained by several key limitations yet to be fully overcome. These include dependency on high-quality datasets, inaccuracies in PTM site localization, challenges in defining reliable negative examples, and difficulties validating predictions at scale. Overcoming these challenges is essential for advancing computational PTM discovery methods.

As experimental techniques for identifying PTMs matured, improvements in computational power and algorithm development gave rise to *in silico* techniques for substrate network discovery. Many PTM databases have been created to curate access to the PTMs found with the techniques covered above, advancing the development of PTM predictive models. Databases such as dbPTM, BioGRID, and PhosphositePlus are a few of the generalized repositories of PTM information across a variety of organisms [3,6,70,71,72]. Specialized databases exist for certain PTMs, including phosphorylation, glycosylation, and ubiquitination [3]. Compiled from literature reviews of PTM discovery, dbPTM contains over 2,777,000 PTM sites from a variety of organisms [6].

Initially, computational techniques arose to annotate the proteome with PTMs, unspecified towards the enzymes responsible. The first foray into computational predictions of PTMs was in 1999, with NetPhos, an artificial feedforward neural network to search for phosphorylation sites [73]. NetPhos was constructed with a couple of hundred substrates, dwarfed by the massive datasets available today, although it performed with a reported sensitivity of 69 to 96% [73]. In the decades since, over 40 other computational tools have been developed to predict phosphorylation sites within the proteome [19]. Many annotation tools exist for other types of PTM, including methylation, S-sulfenylation, palmitoylation, pupylation, ubiquitination, and more [3,20]. For an excellent overview of available PTM annotation methods for proteomic analysis, we direct the reader to Ramazi and Zahiri, 2021 [3]. Most of these tools implement predictive strategies using known sites of PTM activity, and some protein-based context of the sites affected (Table 1). In the case of PTM prediction, the computational approach is one of classification: is the PTM occurring or not? Hence, it is vital that these computational approaches have access to sites of known PTM activity (deemed as positive), and sites where no PTM activity should occur (deemed as negative).

Often, published annotative tools fall short in practical application, as demonstrated by Piovesan et al. (2020), who evaluated seven hydroxylation prediction models and found that none performed better than random on new datasets [90]. A key limitation underlying this poor performance is the difficulty in accurately defining negative training datasets. True negatives are rarely catalogued in PTM repositories and nearly impossible to determine with confidence; given the continual discovery of new modification sites, residues currently labeled as unmodified may, in fact, be false negatives. In the absence of clearly defined negatives, some tools generate them randomly or sample from the unannotated proteome—approaches that risk including unknown positives and thus compromise model reliability [90]. To address this, a few tools have incorporated extensive experimental validation, such as MethylSight, a lysine methylation predictor that demonstrated strong performance (a specificity of 97% and a precision of 63%) through rigorous laboratory confirmation of its predictions [74]. Unfortunately, such experimental follow-up remains uncommon across most predictive platforms.

The advent of deep learning and neural networks has introduced novel, comprehensive methods that annotate the proteome for multiple PTMs rather than focusing on one PTM. Additionally, these tools have moved from utilizing solely short sequential peptides representative of the modification site, to incorporating the entire protein sequence (Table 1). MusiteDeep applies deep learning to annotate the sites of 12 different PTMs through an easily accessible webserver with performance reported through an area under the curve (AUC) range of 0.732–0.990 for the receiver operating characteristic (ROC), and 0.279–0.947 AUC for the precision-recall (PR) across various PTM types [75]. Recently, a multi-label interpretable deep-learning method for PTM prediction-structure version (MIND-S) has been released, an AI-based PTM prediction tool which incorporates multi-label interpretable deep learning method for PTM prediction [86]. This tool employs both protein sequences and the amino acid contact points within the PTM-inducing enzyme–substrate interaction as layers within a fully connected neural network. MIND-S has the capability to annotate 26 different PTMs across the proteome, allowing for widescale batch predictions, with a reported average AUC PR value of 0.701 [86].

With the onset of powerful language models such as ChatGPT (OpenAI, San Fransico, CA, USA), protein language models have emerged as a potential avenue for PTM prediction, where the extensive libraries of known protein sequences are used to disseminate patterns that may be applied to biological function. In 2024, Shrestha et al. introduced PTMGPT2, a suite of models for PTM site identification constructed with ProtGPT2, which uses generative pretrained (GPT) transformer-based models [87]. These models are unsupervised, meaning they do not require labelled datasets of known positive and negative sites for PTMs, first training to learn the general patterns of proteins from a large amount of protein sequences. Next, prompt-based fine tuning utilizes a smaller set of known PTMs to adjust the model so it may apply its learned pattern of proteins to predict where PTMs occur. PTMGPT2 outperformed MuSite deep in N-linked glycosylation on asparagine by 5.62%, and arginine methylation by 12.74%, performing with an overall specificity of 31.25–82.37% and a sensitivity of 27.48–80.78% across various PTM types [87]. Evidently, PTM annotation is rapidly evolving with computational advances, however enzyme-specific annotation remains comparatively underrepresented.

Discovering PTMs in the context of a particular enzyme with computational approaches is relatively less explored; however, some techniques have been developed. PredPhospho utilizes support vector machines (SVMs) and families of kinases to predict sites of phosphorylation specific to that family [76]. Although PredPhospho is not enzyme-specific, but enzyme family-specific, its early development in 2004 represents the beginning of enzyme-specific methods of PTM prediction. This technique uses a compiled dataset sourced from multiple databases, and reportedly performs with an accuracy rate of 76–95% [76]. Other family-specific techniques have been developed, including Quokka, which improves upon PredPhospho, and ASEB, which predicts for families of lysine acetyltransferases, reportedly performing with a ROC AUC of 0.870–0.955 [77,78].

Machine learning (ML) methods have been developed to predict the PTM activity of a handful of specified PTM-inducing enzymes. GPS-PAIL annotates the proteome for the activity of 7 histone acetyltransferases, performing with a reported specificity range of 80–100%, and a variable precision rate of 19.23–100% [79]. The training data employed to generate GPS-PAIL is compiled from the literature, and the model uses a unique group-based prediction system (GPS) algorithm to predict activity [79]. CRPhos is another PTM site predictor, specific to the phosphorylation sites of a defined kinase, reportedly performing with an AUC ROC of 0.91–0.97 [80]. An additional phosphorylation predictor, PhosphoPredict expands to annotate the PTM-induced enzyme activity of 12 kinases, with a reported AUC ROC of 0.889 and an AUC PR of 0.881 [81].

An alternative method of PTM-induced enzyme activity prediction is presented by Lanouette et al., in which computational protein design (CPD) is applied to investigate the KMT SMYD2 [89]. Based off multistate design, a process in which different protein conformational states are considered as inputs, CPD predicts protein sequences with multiple protein backbone templates as input to generate a substrate recognition motif for SMYD2 computationally [89]. The crystal structure of SMYD2 was required for CPD analysis, and the computationally generated motif was verified experimentally, performing with an accuracy rate of 86% [89]. This motif was then applied to identify four potential novel substrates of SMYD2 [89].

A commonality of methods for PTM prediction is the reliance on experimental databases of PTMs for model construction, or the crystal protein structure. In the case of enzyme-specific PTM prediction, this becomes a hinderance as many PTM-inducing enzymes possess just a handful of verified substrates. This is further emphasized by the lack of enzyme-specific PTM prediction methods that are constructed with databases. A potential solution to this problem is to employ the high-throughput method detailed above for the creation of curated, high-confidence datasets for ML techniques, while retaining similar predictive approaches (Table 1). Pre-emptive dataset construction also mediates the issue posed by the lack of negative examples in databases. For the purposes of this review, we will call the process of actively generating experimental data prior to ML creation “hybrid” approaches.

The first detailed example of a hybrid approach was presented in 2009, with SVM-PEPARRAY [82]. A webserver enables users to upload peptide array or microarray sequences along with PTM activity induced by the enzyme studied. The user may select one of two feature representations of the peptide sequences, then an SVM model is fit and finely tuned [82]. Proteome annotation may be completed through the upload function of a list representative of the sites of potential modification within the proteome [82]. As of writing, the SVM-PEPARRAY webserver is inaccessible for further analysis and wet-lab validated performance metrics are unknown.

Another approach of hybrid enzyme specific PTM discovery was presented by Ferrari et al. in 2011 [88]. Sites of protein tyrosine phosphatase 1B (PTP1B) activity are annotated through Bayesian modelling [88]. Peptide microarray analysis was first performed on a number of phopsho-peptides to generate a position specific scoring matrix (PSSM), descriptive of the binding site recognition of PTP1B [88]. This PSSM, along with a distance matrix detailing a weighted protein interaction graph is utilized within Bayesian integration [88]. The resulting model is applied to identify novel substrates of PTP1B phosphatase activity within the proteome, isolating highly ranked substrates that are affiliated with the insulin and epidermal growth factor (EGF) pathways [88]. The ROC AUC was identified to be 0.85 through the application of validation data of approximately 6000 human phosphotyrosine peptides (naturally occurring) [88]. Seven of the predicted substrates implicated in EGF and insulin pathways were tested with a substrate trapping mutant of PTP1B, of which five were confirmed to bind [88].

The peptide optimization with optimal learning (POOL) technique is another hybrid approach, applied to analyze 4′-phosphopantetheinyl transferase (PPTase) [83]. Initially, a naïve Bayes ML model is fit and trained using data determined from a SPOT array experiment [83]. The model iteratively generates new peptides to test with SPOT array for reinsertion prior to another round. This “active learning” process is completed over and over to effectively optimize the predictive capabilities of the model; however, no performance metrics were reported. Although the POOL method was applied to generate peptides that bound to PPTase, the authors state that the trained model may be applied to score substrates within the proteome [83].

A recent hybrid technique employs the ultra-high throughput mRNA display method along with deep learning [84]. The mRNA display utilizes high-throughput RT-PCR, ligation, and translation to generate randomized protein and peptide libraries with trillions of variants [91]. This technique was applied to create a large randomized library with peptides of 11 AAs in length for the exposure to serine dehydratase and cysteine/serine cyclodehydratase [84]. Affected peptides were detected through biotinylation and separated with a streptavidin pulldown, and the remaining sequences were determined through next-generation sequencing to elucidate the peptide sequences [84]. Data were then applied to train deep learning models to predict the enzyme-specific PTM activity, with high confidence (accuracy of 0.993) due to the large dataset of 10^13^ sequences [84]. This impressive method has the capability to perform well on other PTMs; however, it is momentarily limited to PTMs that may be biotinylated.

## 6. Limitations

As discussed, each of the methods present both benefits and disadvantages over one another, but, in general, there are some shared limitations throughout. Throughout the field of enzyme-specific PTM discovery, there exists a lack of high-quality enzyme-specific PTM data. Additionally, as the field of computational-based approaches has continued to develop, the difference in performance metrics reported makes it difficult to accurately compare approaches. This issue is only compounded by the lack of standards in documentation and maintenance, preventing access to older tools so they can be properly benchmarked with the new. As most methods are informed by confirmed sites of PTM activity, the inherent shortage of data presents a significant hurdle, caused by both the lack of reported negatives for use in training data, and the inherent bias within known PTMs due to discovery techniques such as permutation arrays. This issue is further compounded by the absence of site specificity within broad search approaches such as MS, which is prone to PTM localization errors. The general difficulty in validating PTM predictions at scale presents a further issue, as the presence of a proteoform displaying the PTM of interest is dynamic in nature. This explains the limited proteoform-level resolution within all discovery approaches discussed here. The functional proteoform diversity may be misled by the methods discussed in this review and remains a pertinent issue to be addressed as the field advances, particularly so in computational approaches.

## 7. Conclusions and Outlook

Significant strides have been made in enzyme–substrate discovery for PTMs over the past century, driven by chemical, technological, and computational advancements. The integration of experimental techniques, such as top-down proteomics, peptide arrays, and mass spectrometry, with computational tools is crucial for the comprehensive discovery of enzyme–substrate networks. Advancements in high-throughput experimental methodologies, combined with sophisticated computational predictions, are poised to reduce existing biases and accelerate the identification of novel PTMs. A balanced approach leveraging the strengths of both experimental and computational methods will significantly enhance our understanding of functional proteomics.

As chemical and technological advances have grown, so has the rate of PTM discovery. The link of each PTM to the enzymes responsible has proved an increasingly difficult task, due to the complexity of the problem at hand. The individual investigation of PTM-inducing enzymes proves to be a tedious task, which stands to be simplified through computational and analytical techniques. With the advent of improved *in silico* techniques, PTM-inducing enzyme–substrate discovery has accelerated in recent years. Although the field momentarily remains skewed towards well-studied PTMs such as phosphorylation, techniques in artificial intelligence stand primed to transform the landscape greatly. As demonstrated with the hybrid techniques described, the concurrent advance in high-throughput analysis has the potential to reduce bias in PTM discovery. With the continual advancement of computational approaches, advancements in high-throughput and ultra-high-throughput techniques will support enzyme-specific substrate discovery. Uncovering the enzyme–substrate networks of PTM-inducing enzymes is the next logical step in detailing the functional proteome.

## Figures and Tables

**Table 1:** Overview of the features applied in the various ML-based tools for PTM substrate discovery discussed within this review.

Type of Feature	Tool-Specific Details	Tool Name
Sequence-Based	Peptide or fragment of protein, surrounding PTM site	NetPhos [73], MethylSight [74], MusiteDeep [75], PredPhospho [76], Quokka [77], ASEB [78], GPS-PAIL [79], CRPhos [80], PhosphoPredict [81], SVM-PEPARRAY [82], POOL [83], mRNA Display [84]
Sequence-Based	Physicochemical feature vectors	MethylSight [74], CRPhos [85], PhosphoPredict [81], mRNA Display [84]
Sequence-Based	Full protein sequence, PTM site(s) indicated	MIND-S [86], PTMGPT2 [87]
Sequence-Based	Substitution matrix: BLOSUM or PSSM	Quokka [77], ASEB [78], GPS-PAIL [79], PTP1B Bayesian Modelling [88]
Sequence-Based	Recognition motif	Quokka [77]
Sequence-Based	Protein interaction network	PhosphoPredict [81]
Sequence-Based	Predicted secondary structure	MIND-S [86], PhosphoPredict [81]
Structure-Based	Structure of enzyme–substrate complex	CPD [89]
